# Chorea and Tænia Solium

**Published:** 1904-04-23

**Authors:** 


### CHOREA AND TÆNIA SOLIUM.

Dr. James Burnet[1] reports two cases of severe chorea associated with tapeworms. Both patients were females, one aged 17 years and the other 12. In both there was a family history of rheumatism, while the younger one had previously suffered from definite rheumatic manifestations, and showed a dilated heart with a markedly accentuated pulmonary second sound. In the former case 5 grs. of calomel were given, and a mixture of sodium bromide and digitalis ordered. As no improvement ensued, 10 gr. doses of sodium salicylate were given in addition every six hours without any beneficial result. The probability of worms being present was then thought of, and accordingly a capsule containing extrac. Filicis liq. was ordered. This resulted in the passage of a large tapeworm. From this time the patient began to improve, and in a few weeks was perfectly recovered. In the second case a preliminary dose of calomel and $7\frac{1}{2}$-gr. doses of aspirin every four hours having been tried, arsenic was added, but still without success. A capsule containing 30 mms. of ext. Filicis liq. was then given, with the result that a fairly large specimen of *Tænia solium* was passed. The movements subsided almost immediately, and the child got perfectly well, except that the heart remained slightly dilated with accentuation of the pulmonary second sound.

Although mild choreiform movements in children may be associated with the presence of any form of intestinal worm, such violent chorea as occurred in Dr. Burnet's cases must be uncommon with such an association. It appears that the possibility of tapeworms being present should be borne in mind in all cases of chorea which do not prove amenable to usual remedies.

[1] Brit. Jour. Chil. Dis, April 1904.

